# Bortezomib-induced heat shock response protects multiple myeloma cells and is activated by heat shock factor 1 serine 326 phosphorylation

**DOI:** 10.18632/oncotarget.10847

**Published:** 2016-07-26

**Authors:** Shardule P. Shah, Ajay K. Nooka, David L. Jaye, Nizar J. Bahlis, Sagar Lonial, Lawrence H. Boise

**Affiliations:** ^1^ Department of Hematology and Medical Oncology, Winship Cancer Institute of Emory University and the Emory University School of Medicine, Atlanta, GA, USA; ^2^ Department of Pathology and Laboratory Medicine, Emory University School of Medicine, Atlanta, GA, USA; ^3^ Department of Medical Oncology and Hematology, Tom Baker Cancer Center, Calgary, AB, Canada

**Keywords:** myeloma, bortezomib, heat shock, HSF1

## Abstract

Proteasome inhibitors such as bortezomib are highly active in multiple myeloma by affecting signaling cascades and leading to a toxic buildup of misfolded proteins. Bortezomib-treated cells activate the cytoprotective heat shock response (HSR), including upregulation of heat shock proteins (HSPs). Here we inhibited the bortezomib-induced HSR by silencing its master regulator, Heat Shock Factor 1 (HSF1). HSF1 silencing led to bortezomib sensitization. In contrast, silencing of individual and combination HSPs, except HSP40β, did not result in significant bortezomib sensitization. However, HSP40β did not entirely account for increased bortezomib sensitivity upon HSF1 silencing. To determine the mechanism of HSF1 activation, we assessed phosphorylation and observed bortezomib-inducible phosphorylation in cell lines and patient samples. We determined that this bortezomib-inducible event is phosphorylation at serine 326. Prior clinical use of HSP inhibitors in combination with bortezomib has been disappointing in multiple myeloma therapy. Our results provide a rationale for targeting HSF1 activation in combination with bortezomib to enhance multiple myeloma treatment efficacy.

## INTRODUCTION

In 2016, an estimated 30,330 people will be diagnosed with multiple myeloma, a plasma cell malignancy that historically affects older individuals [1]. Unlike most cancers, myeloma cells retain many of the same functions as their normal counterpart, long-lived bone marrow plasma cells, including immunoglobulin secretion [2]. Because plasma cells are constitutive immunoglobulin producers, they are dependent on the proteasome for quality control and survival, and myeloma cells also retain this dependence [2, 3]. Bortezomib is a boronic acid-based proteasome inhibitor which inhibits the β5-subunit of the proteasome. Bortezomib has been a mainstay of myeloma therapy since its Food and Drug Administration (FDA) approval for refractory myeloma in 2003 [4]. The use of bortezomib in combinatorial treatment regimens along with immunomodulatory drugs (IMiDs) has led to a dramatic improvement both in overall survival [OS] (46.6% five-year OS in 2005-2011 versus 29.7% in 1986-1990) and long-term progression-free survival [PFS] (36.0 months median PFS versus 29.7 months with bortezomib plus dexamethasone versus vincristine, doxorubicin, plus dexamethasone [VAD]) [1, 5]. Two additional proteasome inhibitors have recently been FDA-approved for myeloma therapy, highlighting the importance of this class of agents for the treatment of this disease [6–12].

Bortezomib-based regimens have led to remarkable improvement in myeloma patient outcomes. However, maximizing their utility may be difficult because myeloma cells can hijack cytoprotective processes used by normal plasma cells. Myeloma cells are able to counteract the pro-apoptotic effects of bortezomib through upregulation of pro-survival pathways, including the heat shock response (HSR) [13]. The HSR protects healthy cells from stressors such as cold, UV light, and environmental toxins, and myeloma cells activate this cytoprotective mechanism to presumably protect themselves from bortezomib-mediated apoptosis. The HSR is mediated by heat shock proteins (HSPs). HSPs serve a wide variety of functions, but are primarily involved in protein folding and protein homeostasis regulation [14, 15]. HSP inhibitors have been tested in myeloma clinical studies both as single agents and in combination with bortezomib [16, 17]. However, none have been FDA-approved because HSP inhibitors suffer from low potency at clinically relevant levels and an induction in compensatory HSPs [18, 19]. In addition, which HSPs are most critical to mounting a robust HSR is unknown. To counteract this, one strategy is to treat patients with multiple HSP inhibitors, a strategy limited by the presence of over 97 HSP-encoding genes [20]. Therefore, inhibition of bortezomib-mediated HSP induction may require dozens of inhibitors and is not a viable therapeutic approach.

Another strategy is to inhibit multiple HSPs simultaneously by targeting the master transcription factor of the HSR, Heat Shock Factor 1 (HSF1). Under baseline conditions, HSF1 is in an inactive cytoplasmic heterotetramer with HSP40, HSP70, and HSP90 [21]. Maintenance of this heterotetramer is controlled by constitutive post-translational modifications (PTMs) such as phosphorylation of HSF1 at serine 303 (pS303) and pS307 [22]. Upon proteotoxic stress such as proteasome inhibition, the HSR is induced, leading to dissociation of the inactive heterotetramer, HSF1 trimerization and nuclear translocation, and binding to the heat shock element (HSE) of HSP genes [23]. HSF1 pS419, pS230, pS320, and pS326, among other modifications, have been reported to positively regulate HSF1 activity [24–29]. During attenuation of the HSR, HSF1 exits the nucleus, and is either degraded or returns to its inactive state [30].

Here, we show that HSF1 knockdown sensitizes myeloma cells to bortezomib treatment. In addition, we demonstrate that targeting HSF1 is a more effective therapeutic approach than targeting multiple HSPs. Therefore, targeting HSF1 activation and associated bortezomib-induced PTMs is a potential therapeutic approach. We further demonstrate that bortezomib induces phosphorylation of HSF1 on serine 326. Together, these data provide evidence that in order to enhance the efficacy of proteasome inhibition in myeloma treatment, targeting HSF1 is an effective therapeutic strategy.

## RESULTS

**Figure 1:**
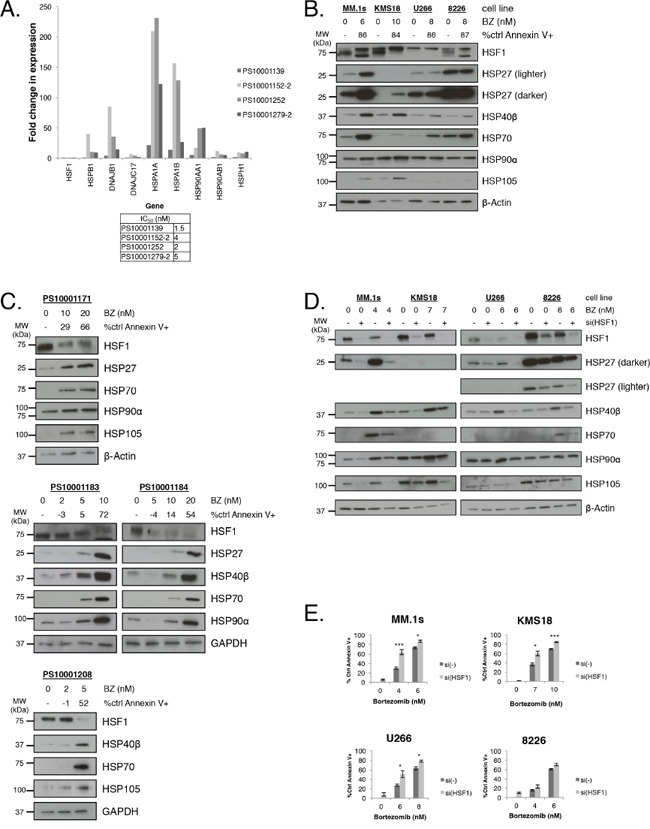
Bortezomib induces HSP expression in multiple myeloma cells, and HSF1 silencing sensitizes multiple myeloma cells to bortezomib treatment **A.** CD138+ cells were purified (>90%) from freshly isolated myeloma patient samples and treated with bortezomib for 24h. Cells were collected at 12h for qRT-PCR gene expression analysis and analyzed at 24h for apoptosis. Gene expression is expressed relative to untreated cells and normalized to GAPDH endogenous control. A table lists bortezomib IC_50_ values. **B.** Myeloma cell lines were treated with bortezomib for 24h. Protein lysates were collected at 12h for western blot analysis and cells were analyzed at 24h for apoptosis. Apoptosis was measured by Annexin V and PI staining and flow cytometry. Data are representative of three independent experiments. Western blot images have been cropped for presentation clarity. **C.** CD138+ cells were purified (>90%) from freshly isolated myeloma patient samples and treated with bortezomib for 24h. Protein lysates were collected at 12h for western blot analysis and cells were analyzed at 24h for apoptosis. Western blot images have been cropped for presentation clarity. **D.** HSF1 was silenced in myeloma cell lines for 24h and cells were treated with bortezomib for an additional 24h. Protein lysates were collected afterward for western blot analysis. Data are representative of four independent experiments. Western blot images have been cropped for presentation clarity. **E.** Experimental setup was as described in (D). Bortezomib-induced apoptosis was measured by Annexin V and PI staining and flow cytometry. P-value is calculated by paired t-test. (*P<0.05, **P<0.01, ***P<0.001)

Bortezomib-treated myeloma cell lines induce a cytoprotective HSR, characterized by HSP induction and HSF1 mediates this response. Therefore, we wanted to determine whether bortezomib treatment of myeloma patient samples led to HSP gene expression upregulation. RNA was extracted from isolated CD138+ cells from four different myeloma patients following bortezomib treatment (Figure 1A). cDNA was probed for changes in HSP and HSF1 gene expression using qPCR. Bortezomib did not lead to HSF1 gene expression induction. This finding is not surprising because HSF1 expression and activity are regulated at the post-transcriptional level [22, 24, 29, 31–36]. Consistent with previous studies, HSP gene induction was observed in every patient sample and though there was a variable induction pattern between patient samples, HSPA1A was consistently the most upregulated gene followed by HSPA1B. Both of these isoforms code for HSP70. In addition, strong HSP90AA1 (HSP90α) and DNAJB1 (HSP40β) induction was observed.

We then wanted to characterize HSP and HSF1 protein expression before and after bortezomib treatment in four cell lines: MM.1S, KMS18, U266, and RPMI-8226 [8226] (Figure 1B). Consistent with previous findings, bortezomib treatment resulted in the induction of the HSR in all four lines, however the responses were somewhat varied. MM.1S cells showed strong HSP27, HSP40β, HSP70, and HSP105 induction. KMS18 cells showed strong HSP27, HSP40β, and HSP105 and modest HSP70 induction. U266 cells showed strong HSP40β and HSP70 induction while HSP105 was not detected. 8226 cells showed strong HSP40β and modest HSP70 induction and HSP105 was not detected. Baseline HSP90α levels were high in all four lines and none showed strong induction of HSP90α. Notably, baseline HSP27 and HSP70 levels were higher in 8226 cells than in the other cell lines. Also, though HSP induction varied between cell lines, none showed an increase in HSF1 expression. The observed HSF1 gel shift upon bortezomib treatment is consistent with HSF1 post-translational modification. We also probed for HSP and HSF1 expression in bortezomib-treated isolated CD138+ cells from four different myeloma patients (Figure 1C). Consistent with the results in cell lines, bortezomib induced various HSP and did not increase HSF1 expression.

Since HSPs are cytoprotective, a strategy to enhance bortezomib-mediated apoptosis is to reduce HSP induction. Previous studies have concluded that single HSP knockdown may not induce lethality in myeloma and as seen above, bortezomib leads to the induction of a variable pattern of multiple HSPs. Therefore, one approach to enhance bortezomib-mediated apoptosis is to target multiple HSPs either individually or simultaneously. However, identifying and targeting the correct HSP(s) has proven to be a challenge due to the variability observed in the HSR in different samples (Figure 1B-1C). Therefore, we used siRNA to knock down HSF1 (Figure 1D). We treated the four cell lines with HSF1 siRNA and bortezomib. HSF1 knockdown led to a decrease in bortezomib-mediated HSP induction to various degrees, with the exception of HSP90α, which showed minimal decrease in protein expression. HSF1 siRNA treatment resulted in minimal induction of cell death while bortezomib treatment resulted in cell-line and dose-dependent moderate to high apoptosis (Figure 1E). However, with an HSF1 siRNA and bortezomib combination, we observed a greater than additive apoptotic effect with MM.1S and KMS18 cells, an additive effect with U266 cells, and no effect with 8226 cells. Therefore, targeting the global response instead of individual HSPs may be a more effective means to sensitize myeloma cells to proteasome inhibition.

**Figure 2:**
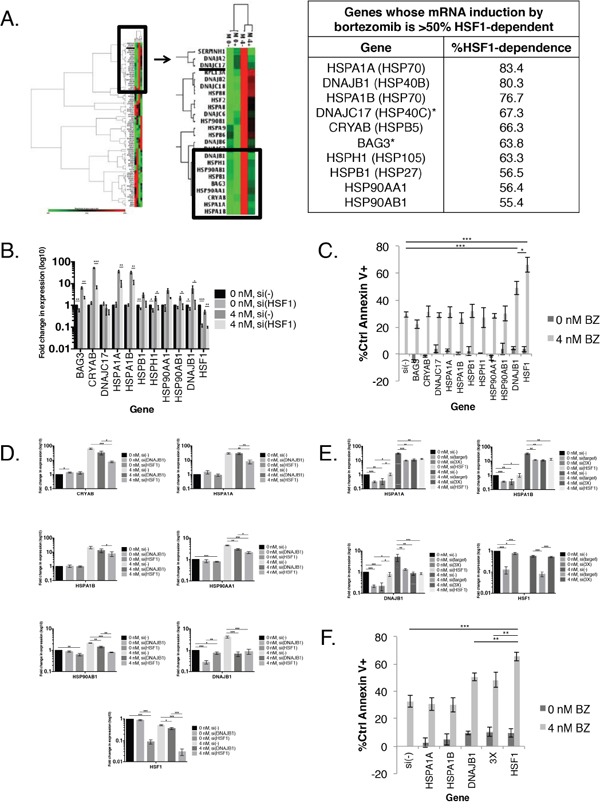
In combination with bortezomib treatment, HSF1 silencing is more effective than HSP silencing at HSR downregulation **A.** (Left) MM.1S cells were treated with a non-silencing control (−) or HSF1 (+) siRNA for 24h followed by 0 or 4 nM bortezomib for an additional 24h. RNA was extracted afterward from whole cell lysates, reverse transcribed to cDNA, and probed for changes in gene expression using the QIAGEN© Human Heat Shock Array qPCR Panel. Gene expression is expressed relative to MM.1S(−), 0 nM and normalized to the mean of five housekeeping genes (B2M, HPRT1, RPL13A, GAPDH, and ACTB). Green indicates lower expression, black indicates no change, and red indicates higher expression. (Right) A table listing all genes whose bortezomib-induced mRNA induction is >50% HSF1-dependent. **B.** Independent confirmation of bortezomib-induced HSF1-dependent genes. Experimental setup was as described in (A). Gene expression is expressed relative to untreated cells and normalized to GAPDH endogenous control. Data are presented as the mean±s.e. of three independent experiments. **C.** MM.1S cells were treated with a non-silencing control [si(−)] or HSP or HSF1 siRNA for 24h followed by 0 or 4 nM bortezomib for an additional 24h. Cells were analyzed at 48h for apoptosis. Apoptosis was measured by Annexin V and PI staining and flow cytometry. Data are presented as the mean±s.e. of three independent experiments. **D.** MM.1S cells were treated with a non-silencing control [si(−)] or single gene (DNAJB1 or HSF1) siRNA for 24h followed by 0 or 4 nM bortezomib for an additional 24h. RNA was extracted from whole cell lysates, reverse transcribed to cDNA, and probed for changes in gene expression. Gene expression is expressed relative to untreated cells and normalized to GAPDH endogenous control. Data are presented as the mean±s.e. of three independent experiments. **E.** MM.1S cells were treated with a non-silencing control [si(−)], single gene (HSPA1A, HSPA1B, DNAJB1, HSF1) or combination (3X: HSPA1A + HSPA1B + DNAJB1) siRNA for 24h and 0 or 4 nM bortezomib for an additional 24h. RNA was extracted at 48h from whole cell lysates, reverse transcribed to cDNA, and probed for changes in gene expression. Gene expression is expressed relative to untreated cells and normalized to GAPDH endogenous control. Data are presented as the mean±s.e. of three independent experiments. **F.** Setup was as described in (E). Bortezomib-induced apoptosis was measured by Annexin V and PI staining and flow cytometry. P-value is calculated by paired t-test. (*P<0.05, **P<0.01, ***P<0.001)

To determine if knockdown of expression of one or more HSP was responsible for the increased apoptosis observed with HSF1 knockdown, we used an 84-gene HSP gene expression array (Figure 2A). We treated MM.1S cells with bortezomib and HSF1 siRNA and probed for changes in HSP gene expression. We found several patterns of gene expression in this 84-gene panel, including genes that were induced by HSF1 silencing in the absence or presence of bortezomib (Supplementary Table 1). However we focused on genes that were induced by bortezomib (Figure 2A, zoomed region). Of the 17 genes induced at least twofold by bortezomib, the induction of 10 was inhibited by at least 50% by HSF1 silencing (Figure 2A). We independently confirmed these genes as HSF1-dependent by qRT-PCR (Figure 2B).

Next, to determine if one or more HSP was responsible for the observed HSF1 protective effect, we compared the effect of HSF1 silencing to silencing specific HSPs on bortezomib-induced apoptosis (Figures 2C and Supplementary Figure 1). Only the silencing of DNAJB1 (HSP40β) showed a significant increase in bortezomib-induced apoptosis when compared to a control siRNA. However, the apoptosis seen with DNAJB1 siRNA and bortezomib was significantly lower than that of HSF1 siRNA and bortezomib. Therefore, no individual HSP can account for HSF1′s observed protective effect. To further explore DNAJB1′s role in the HSR, we treated MM.1S cells with DNAJB1 or HSF1 siRNA with bortezomib and probed for various HSP genes (Figure 2D). DNAJB1 knockdown led to significant reduction of HSP90AA1 and HSP90AB1 bortezomib-mediated induction, but not nearly to the same level as HSF1 knockdown. DNAJB1 knockdown and HSF1 knockdown resulted in similar reduction of DNAJB1 induction. However, DNAJB1 knockdown did not lead to reduction of CRYAB, HSPA1A, and HSPA1B gene induction. Thus while DNAJB1 knockdown influences the HSR, which likely accounts for its protective effects, it does not fully replicate the activity of HSF1.

Our data suggest that silencing HSF1 sensitizes cells to bortezomib through its regulation of multiple HSRs. Therefore, we next tested if simultaneous knockdown of multiple HSP genes could replicate the apoptotic or regulatory effects of HSF1 knockdown upon bortezomib treatment. We silenced the three most HSF1-dependent HSP genes as listed in Figure 2A; HSPA1A, HSPA1B, and DNAJB1 (simultaneous knockdown of all three = 3X), and determined the effect on gene expression (Figure 2E) and apoptosis (Figure 2F). At the gene expression level, there was no evidence that individual HSPA1A and HSPA1B knockdown had any regulatory effect on the expression of other HSPs. Silencing of all three HSPs did not significantly reduce HSP gene induction levels below individual siRNA treatment. Additionally, inducible HSP levels remained significantly above that of HSF1 siRNA. Silencing of all three HSPs with bortezomib resulted in higher apoptosis than bortezomib alone, HSPA1A siRNA with bortezomib, and HSPA1B with bortezomib. Apoptosis was similar to DNAJB1 siRNA with bortezomib, and lower compared to HSF1 siRNA with bortezomib. Taken together these data suggest that expression of the three most bortezomib-induced HSF1-dependent HSP genes cannot account for the survival effects of HSF1 knockdown. These findings imply that targeting HSF1 would be a more effective approach than targeting HSPs to enhance proteasome inhibitor activity.

**Figure 3:**
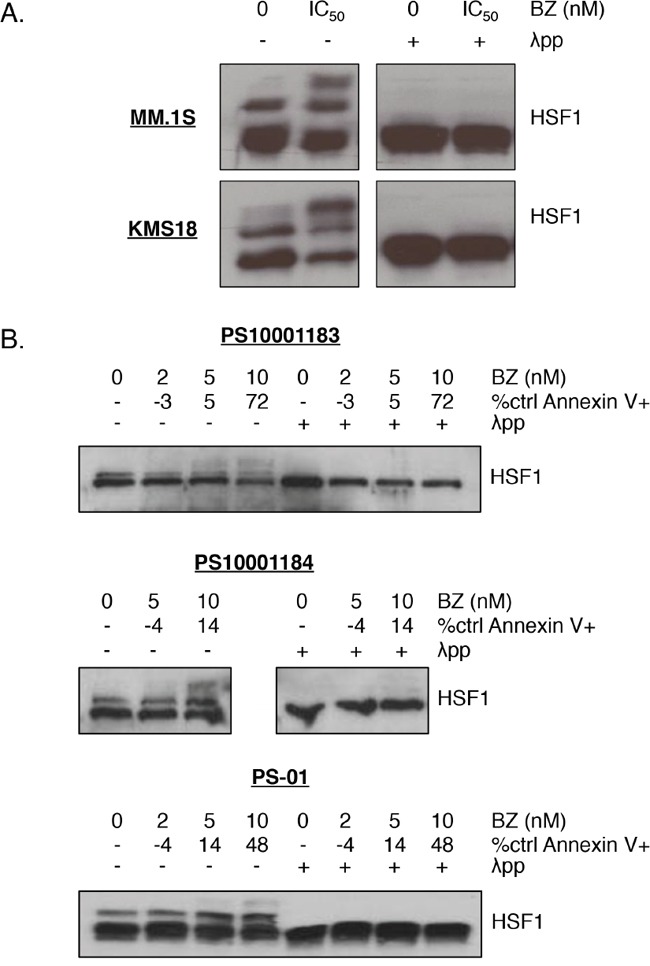
HSF1 is phosphorylated upon bortezomib treatment in multiple myeloma cells **A.** MM.1S and KMS18 cells or **B.** CD138+ cells from freshly isolated patient samples were treated with bortezomib (MM.1S: 5 nM, KMS18: 8 nM) for 24h. Protein lysates were collected at 12h for western blot analysis and cells were analyzed at 24h for apoptosis. Phos-Tag™ western blotting was performed on prepared lysates followed by HSF1 detection. (λ phosphatase was used to determine which bands were due to phosphorylation.) Bortezomib-induced apoptosis at 24h is indicated by percent control Annexin V+. Cell line data is representative of seven independent experiments. Western blot images have been cropped for presentation clarity.

Currently, there are no HSF1 inhibitors that are FDA-approved or even in clinical trials, and published data for many inhibitors raise questions ranging from specificity to efficacy [29, 37]. Therefore, we pursued an approach targeting HSF1 activation, and specifically, PTMs that mediate activation. Based on prior studies of HSF1 activation, we initially focused on bortezomib-induced changes in phosphorylation. To demonstrate that HSF1 is modified by phosphorylation, we used Phos-Tag™ electrophoresis [38]. We employed this technique to detect HSF1 constitutive and bortezomib-induced phosphorylation patterns in MM.1S and KMS18 cells. In these cell lines, under baseline conditions, there are two bands: one showing unphosphorylated HSF1 and one, which is sensitive to λ phosphatase treatment, demonstrating constitutive HSF1 phosphorylation (Figure 3A). Bortezomib treatment led to the presence of an HSF1-inducible phosphorylation band while unphosphorylated and constitutively phosphorylated HSF1 expression decreased. In three different patient samples, bortezomib treatment also led to the presence of an inducible HSF1 phospho-species (Figure 3B). Two of these samples also showed strong bortezomib-inducible HSP upregulation (Figure 1C).

**Figure 4:**
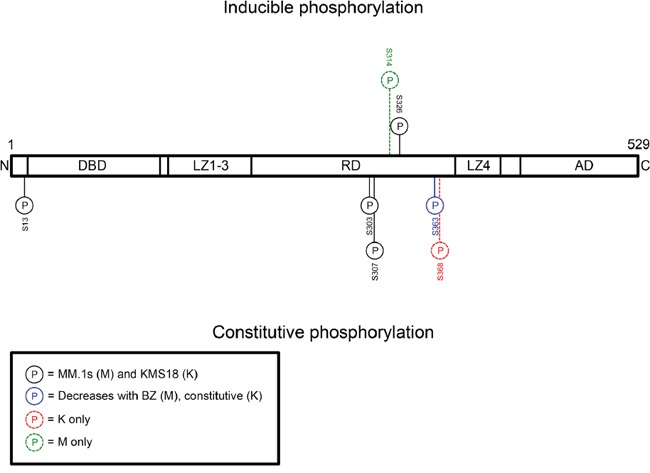
Phosphoproteomics reveals that HSF1 serine 326 is a bortezomib-inducible phosphorylation site and serine 303 is a constitutive phosphorylation site MM.1S and KMS18 cells were treated with bortezomib for 9h and cells were lysed. Immunoprecipitated or gel excised HSF1 was sent to the Emory University Proteomics Core for phosphoproteomics analysis. Detected constitutive and inducible PTMs are represented here.

**Figure 5:**
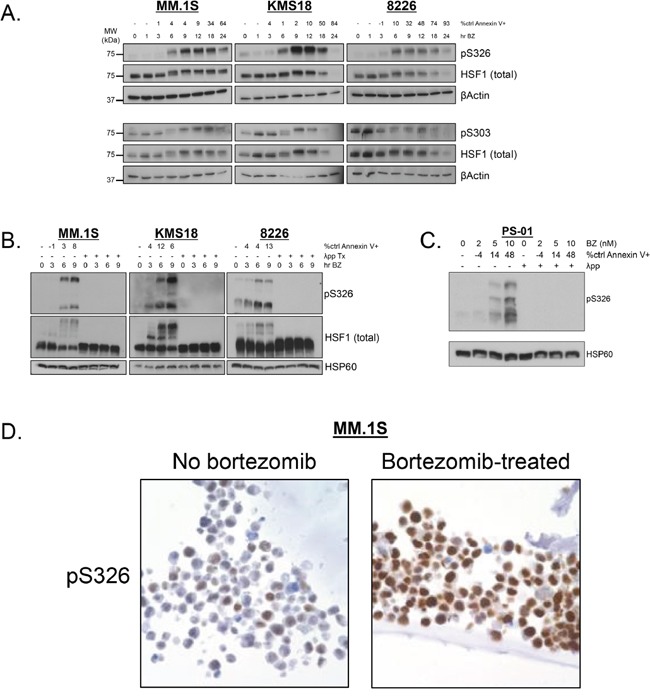
Phospho-specific antibodies confirm that HSF1 serine 326 is a bortezomib-inducible phosphorylation site and serine 303 is a constitutive phosphorylation site **A.** MM.1S, KMS18, and 8226 cells were treated with bortezomib (MM.1S: 5 nM, KMS18: 10 nM, 8226: 8 nM) for up to 24h and lysed at various timepoints. Bortezomib-induced apoptosis is indicated by percent control Annexin V+. Western blot analysis was performed on prepared lysates. Western blot images have been cropped for presentation clarity. **B.** MM.1S. KMS18, and 8226 cells were treated with bortezomib (MM.1S: 5 nM, KMS18: 10 nM, 8226: 8 nM) for up to 9h and lysed at various timepoints. Bortezomib-induced apoptosis is indicated by percent control Annexin V+. Phos-Tag™ western blotting was performed on prepared lysates. (λ phosphatase was used to determine which bands were due to phosphorylation.) Western blot images have been cropped for presentation clarity. **C.** CD138+ cells from freshly isolated patient samples were treated with bortezomib for 24h and cells were lysed at 9h. Bortezomib-induced apoptosis at 24h is indicated by percent control Annexin V+. Phos-Tag™ western blotting was performed on prepared lysates. Western blot images have been cropped for presentation clarity. **D.** MM.1S cells were treated with bortezomib for 9h and fixed. Slides were stained with pS326 (1:2000 dilution), counterstained with hematoxylin, and visualized by immunocytochemistry.

Next, we wanted to identify HSF1 phospho-species detected by Phos-Tag™. Therefore, we performed phosphoproteomic analysis to detect HSF1 phospho-species with and without bortezomib treatment in MM.1S and KMS18 (Figure 4). One inducible site, phosphoserine (pS) 326 was detected in both lines. Constitutive pS13, pS303, pS307, and pS363 was observed in both lines while constitutive pS368 was seen in KMS18 but not MM.1S cells. Notably pS13 and pS368 are previously undescribed HSF1 phosphorylation sites, and bortezomib treatment decreased pS363 expression in MM.1S cells. Inducible pS314 was observed in MM.1S but not KMS18 cells. Using these data, we tested available HSF1 phopshoantibodies, pS326 and pS303. We treated MM.1S, KMS18, and 8226 cells with bortezomib for 24h, collected protein lysates at various timepoints, and probed for pS326 and pS303 expression (Figure 5A). For all three lines, pS326 expression was minimally present at 0h and increased at each timepoint until 9h in MM.1S and KMS18 cells and 6h in 8226 cells. pS326 expression decreased to near baseline levels by 24h. This finding confirmed phosphoproteomics studies of MM.1S and KMS18 cells that detected S326 as a bortezomib-inducible phosphorylation site. Also, in MM.1S and KMS18 cells, there was a stronger pS326 peak than in 8226 cells, and taken together with data shown above, provides evidence of a more robust bortezomib-induced HSR in MM.1S and KMS18 than 8226 cells. For pS303, we confirmed a constitutive phosphorylation pattern in MM.1S, KMS18, and 8226 cells. However, pS303 expression decreased with bortezomib treatment in 8226 cells. This differential expression pattern may be due to the lack of a strong HSR in 8226 cells. As a result, 8226 HSF1 modifications associated with HSR negative regulation may not be as active. In addition, we used Phos-Tag™ and available HSF1 phosphoantibodies to determine the contribution of pS326 to total HSF1 inducible phosphorylation (Figure 5B). We observed that phosphorylation at serine 326 is responsible for HSF1 inducible phosphorylation. In agreement with data shown above, pS326 increases in all three lines, with a 9h peak in MM.1S and KMS18 cells and 6h in 8226 cells. Additional phosphorylation events, as visualized by the intermediate bands showing phospho-species in membranes probed for total HSF1, precede inducible pS326 phosphorylation. However, their identity could not be determined. HSP60 is a mitochondrial HSP and known as a “housekeeping protein”. Here, it is used as a loading control. In a patient sample, pS326 is also responsible for HSF1 inducible phosphorylation (Figures 5C and 3B). In addition, we analyzed constitutive and inducible pS326 expression in MM.1S cells by immunocytochemistry (Figure 5D). Cells were stained with pS326 and counterstained with hematoxylin. We observed that bortezomib leads to a strong induction of nuclear pS326.

## DISCUSSION

Bortezomib has been a mainstay of myeloma therapy since its FDA approval in 2003 and is commonly used in combination with cyclophosphamide, melphalan, prednisone, IMiDs, and dexamethasone [39]. Bortezomib-based regimens have significantly improved patient survival, but bortezomib resistance is common and can lead to relapse [40]. Here, we confirmed that bortezomib treatment leads to upregulation of the cytoprotective HSR (Figure 1A-1C). Strategies to downregulate the HSR in myeloma have not been successful in clinical trials. For example, HSP90 inhibitors have been tested in clinical trials but have not been effective in myeloma [16, 17, 41]. Interestingly, our data show that bortezomib treatment did not lead to HSP90 induction in any of the four cell lines tested (Figure 1B). This result differs from previously published reports. However these early studies used very high concentrations of bortezomib that resulted in only modest changes at the protein level [13]. Therefore, one of the reasons why HSP90 inhibition may not be sufficient in combination with bortezomib is because myeloma cells have constitutively high HSP90 protein expression that does not significantly increase with bortezomib treatment.

Instead of attenuating the bortezomib-induced HSR with multiple HSP inhibitors, we hypothesized that knocking down HSF1 would inhibit bortezomib-induced upregulation of the HSR and sensitize myeloma cells to bortezomib treatment (Figure 1D). HSF1 knockdown led to inhibition of the HSR in all four cell lines tested, and bortezomib sensitization in three (Figure 1E). The fourth line, 8226, had higher baseline levels of HSP27 and 70 than the other cell lines, thus leading to the observation that HSF1 knockdown may not have as strong of an effect on survival because the bortezomib-induced HSR is more robust in the other cell lines compared to 8226. This result is consistent with our previous findings demonstrating that 8226 is more efficient at IgL secretion than MM.1S, which suggests that IgL production does not contribute as heavily to proteasome load in this cell line [3]. Clinical bortezomib resistance may arise when patient myeloma cells that were once responsive to bortezomib deregulate the HSR. This could lead to an increase in basal HSP levels and loss of bortezomib sensitivity.

Since HSPs have proven to be targetable by small molecule inhibitors, we next determined whether a single or multiple HSPs were responsible for HSF1-depdendent survival following proteasome inhibition. Consistent with the HSR being a systemic response to stress, we demonstrated that 9 HSPs were upregulated in an HSF1-dependent fashion (Figure 2A). It is not surprising, therefore, that silencing of any single HSP or even the three most HSF1-dependent HSPs was not as effective as silencing HSF1 (Figure 2C-2F). Taken together, these data suggest that targeting HSF1 would be a more promising approach to bortezomib sensitization than targeting individual or even multiple HSPs. Interestingly, while several small molecule inhibitors of HSF1 have been reported, most are not specific for HSF1 [29, 37, 42–46]. Pre-clinical studies using HSF1 inhibitors alone or in combination with existing treatments such as bortezomib are limited and it remains unclear if these inhibitors can be developed into therapeutic agents [47, 48]. In addition, previous studies have pointed to HSF1 activation as a critical component of the cellular response to proteasome inhibition [49, 50]. Therefore we focused on targeting HSF1 activation upon proteasome inhibition in myeloma cells.

The activation of HSF1 occurs through post-translational modifications that allow this transcription factor to be released from HSP binding, move to the nucleus, bind DNA, and activate transcription from HSE-containing promoters. We showed that HSF1 is phosphorylated upon bortezomib treatment in cell lines and patient samples and identified and confirmed an inducible phosphorylation site, serine 326 (Figures 3-4). We also confirmed that bortezomib treatment leads to nuclear pS326 accumulation (Figure 5). pS326 has been shown to positively regulate HSF1 transactivation on HSE-containing promoters in HeLa cervical carcinoma cells and MDA-MB-231 breast cancer cells [51, 52]. In addition, hyperphosphorylation of serine 326, which is upregulated in breast cancer compared with its normal counterparts, has been used as a biomarker to indicate HSF1 activation in immortalized primary mammary epithelial tumor cells [53, 54]. DNA-PK, ERK1/2, MEK, mTOR, and PI3K have been shown to be responsible for serine 326 phosphorylation in various systems [24, 27, 51, 53, 55, 56]. Knowledge of which kinase is responsible for this phosphorylation event upon bortezomib treatment in myeloma could facilitate development of effective kinase and proteasome inhibitor combination treatments. These treatments could dampen the bortezomib-induced HSR and increase myeloma cell apoptosis. We have initiated studies to determine the bortezomib-inducible HSF1 kinase and our preliminary data show that the responsible kinase is not JAK, JNK, or MEK (S.P.S. and L.H.B., unpublished data, April 2016).

Future studies should explore the role of other HSF1 phosphorylation sites in myeloma beyond serine 326, including sites of constitutive phosphorylation. Our data show constitutive phosphorylation on serine 13, 303, 307, and 363, and 368. In agreement, others have shown constitutive phosphorylation on serine 303 (catalyzed by GSK3α/β), 307 (ERK1/2, JNK), and 363 (JNK, PKC) in other systems [22, 24, 31, 35]. Serine 13 and 368 are previously undescribed sites and require further exploration with regard to their role in HSF1 activation. Promoting constitutive phosphorylation events could keep HSF1 from becoming fully activated, thus leading to a downregulated HSR. Therefore, knowledge of constitutive phosphorylation events and their respective kinases could lead to additional types of combinatorial treatments, such as pairing phosphatase inhibitors with proteasome inhibitors.

Kinase and proteasome inhibitor combination treatments are currently being studied in myeloma, including combining aurora-A, Chk1, CDK, Akt, MEK, mTOR, PI3K, and p38 inhibitors with bortezomib [57, 58]. Interestingly, the latter five kinases have been reported to phosphorylate HSF1 [29]. Furthermore, a recent study found that bortezomib treatment increases Pim half-life by prevention of Pim proteasomal degradation and therefore, the inclusion of a Pim kinase inhibitor in a bortezomib-based regimen could be effective in myeloma treatment [59]. In addition to phosphorylation, HSF1 PTMs include acetylation and sumoylation. A more detailed understanding of these modifications could provide rationale to test, for example, acetylase/deacetylase inhibitors and SUMOylation inhibitors in combination with bortezomib. For example, SIRT1, an NAD+-dependent deacetylase, has been reported to aid in HSF1 binding to HSE-containing promoters of HSP genes [60, 61]. Therefore, a SIRT1 inhibitor could potentially downregulate the bortezomib-induced HSR.

The data presented in this study show that myeloma cells activate the HSR in response to bortezomib and that targeting HSF1 can downregulate the HSR and sensitize cells to bortezomib treatment. Here, we provide a rationale for pairing bortezomib with an HSF1 inhibitor or drugs that target HSF1 PTMs to enhance the efficacy of bortezomib-based treatment regimens. This novel therapeutic strategy could lead to improved progression-free and overall survival for myeloma patients.

## MATERIALS AND METHODS

### Cell lines

The MM.1S cell line was obtained from Dr. Steven Rosen (City of Hope, Duarte, CA) and Dr. P. Leif Bergsagel (Mayo Clinic, Scottsdale, AZ) provided the KMS-18 cell line. RPMI-8226 (8226/S) and U266 cell lines were purchased from American Type Culture Collection (Manassas, VA). Cells were cultured as previously described [62]. MM.1S and 8226 cell lines were tested and authenticated by sequencing. KMS18 cell line was tested and authenticated by flow cytometry. U266 was not authenticated after purchase; however, phenotypic analysis is consistent with known features for this line, e.g., CCND1 overexpression and BRAF activation.

### siRNA and bortezomib treatment

siRNA was obtained from Dharmacon RNA Technologies (GE Healthcare, Little Chalfont, United Kingdom), selecting the ON-TARGETplus SMARTpool duplexes as the RNAi-specific technology platform. ON-TARGETplus Non-targeting Control Pool was used as a control. 48h viability after ON-TARGETplus Non-targeting Control Pool electroporation was greater than 90% for MM.1S, KMS18, and U266 and greater than 75% for 8226 (data not shown). Cells were transfected using the Amaxa Nucleofector II (Lonza Group, Basel, Switzerland). The following cell lines, reagents, and programs were used: MM.1S: V reagent, program O-023; KMS18: C, T-001; U266: R, X-005; 8226: V, G-015. The following oligonucleotides were used: ON-TARGETplus Non-targeting Control Pool: D-001810-10-20 and ON-TARGETplus SMARTpool: L-009743-00-0005 (CRYAB), L-012735-01-0005 (DNAJB1), L-021141-01-0005 (DNAJC17), L-012109-00-0010 (HSF1), L-005168-00-0005 (HSPA1A), L-003501-00-0005 (HSPA1B), L-005186-00-0005 (HSPCA [HSP90AA1]), L-005187-00-0005 (HSPCB [HSP90AB1]), L-005269-00-0005 (HSPB1), and L-004972-00-0005 (HSPH1). Bortezomib was obtained from LC Laboratories (Woburn, MA).

### Flow cytometry cell death detection

Cells were collected at indicated timepoints. 1.0×10^5^-2.5×10^5^ million cells were washed with 1X phosphate buffered saline (PBS) and resuspended in 500 μL FACS buffer (1% BSA in PBS containing 0.01% sodium azide) containing BioVision 1001-1000 Annexin V-FITC (BioVision, San Francisco, CA) and 1 mg/ml propidium iodide (Sigma-Aldrich, St. Louis, MO). Cell death was then measured with a BD FACSCanto II as previously described [63]. Data were analyzed using FlowJo software (TreeStar, Ashland, OR).

### Immunoblotting

Protein lysate preparation and western blotting were performed as previously described with the following change [62]. PVDF membranes were used and membranes were pre-wet in methanol for two minutes and then incubated in transfer buffer for five minutes. The following primary antibodies were used: rat anti-HSF1 mAb (Enzo Lifesciences, Farmingdale, NY), rabbit anti-HSP27 pAb (Enzo), rabbit anti-DNAJB1/HSP40β pAb (Enzo), rabbit anti-DNAJC17/HSP40C pAb (Abcam, Cambridge, United Kingdom), mouse anti-HSP70/72 mAb (Enzo), rat anti-HSP90α mAb (Enzo), mouse anti-HSP90β mAb, rabbit anti-HSP105/110 pAb (Enzo), rabbit anti-HSF1 phospho-serine (pS) 326 (Abcam), and rabbit anti-HSF1 pS303 (Abcam). The following secondary antibodies were used: ECL Rabbit IgG HRP-linked whole Ab (from donkey) (GE Healthcare), ECL Mouse IgG HRP-linked fragment Ab (from sheep) (GE Healthcare) [for all mouse antibodies except anti-HSP90β], goat anti-mouse IgG HRP (PerkinElmer Life Sciences, Boston, MA) [for anti-HSP90β], and goat anti-rat IgG HRP (Santa Cruz Biotechnology, Santa Cruz, CA).

### Patient samples

A patient sample diagnostics table is provided (Table 1). Ficoll isolated buffy coat from myeloma patient bone marrow aspirates were collected and washed with RPMI 1640 complete medium. CD138+ plasma cells were isolated using CD138 microbeads and MACS Columns as per manufacturer's instructions (Miltenyi Biotec, Bergisch Gladbach, Germany), placed in RPMI 1640 complete medium, and bortezomib-treated at indicated concentrations. All samples were collected from patients who gave prior written consent as per an Institutional Review Board-approved protocol.

**Table 1:** Patient Sample Clinical Diagnostics

Sample	Diagnostic sample	Analysis performed	Age	Sex	ISS stage	CTG	FISH	Prior lines	LEN ref	BTZ ref	CFZ ref	POM ref
10001139	Myeloma	qPCR	61	M	1	46,XY[20]	None	5	Yes	Yes	No	No
10001152-2	Myeloma	qPCR	65	M	3	45,X,-Y[3]/46,XY[26]	gain of 1q, monosomy 13 and 17, del (17p)	3	Yes	Yes	Yes	Yes
10001252	Myeloma	qPCR	69	F	3	46,XX,del(16)(q22)[9]/46,XX[13]	gain of IgH; monosomy 13, t(4;14)	0	No	No	No	No
10001279-2	Myeloma	qPCR	42	F	3	47-49,XX,+1, dic(1;16)(p12;q24),add(8)(p23),t(11;14)(q13;q32),t(13;18)(q14;q21.3),add(17)(p11.1),-19,+2-4mar [cp14]/46,XX[6]	gain of 1q, gain of 13q, t(11;14)	5	Yes	Yes	No	No
10001171	Myeloma	Western	68	M	1	55,XY,t(1;17)(q21;q21),add(4)(p16),+5,+7,+9,+11,+15,+15,-16,+19,+21,+21,+mar[4]/46,XY[29]	trisomy 7, 9, 11	2	No	No	No	No
10001183	Myeloma	Western, Phos-Tag Western	54	F	Unk	48-51,X,-X,del(1)(q32),+3,der(3)add(3)(p21)t(1;3)(q27;q25),+9,+11,add(18)(p11.2),+20,+2-3mar[cp4]/46,XX[16]	gain of IgH, trisomy 3, 9, 11	3	Yes	Yes	No	No
10001184	EMD	Western, Phos-Tag Western	64	F	1	46,XX[30]	trisomy 9	3	Yes	Yes	No	No
10001208	Myeloma	Western	71	M	3	54-59,Y,der(X) t(X;11)(p22.1;q13),del(2)(p13),+3,der(3)t(1;3)(q21;p25),+4,+5,add(5)(q13),+7,add(8)(p11.2)x2,+9,del(10)(q22q24), del(11)(p13p14),del(13)(q12q22),+15,add(15)(q22),+17,add(17)(p12),+18,+19,add(20)(p13),+21,+21,+21,del(22)(q11.2),+2-4mar[cp16]/46,XY[4]	gain of 1q, loss of IgH, monosomy 13, del 13q, del (17p), trisomy 3,7,9,11,17	2	Yes	Yes	No	No
01	Myeloma	Phos-Tag Western	54	M	2	Unk	t(4;14); del 17p	3	Yes	Yes	Yes	Yes

EMD: extramedullary myeloma; M: Male; F: Female; ISS: International Staging System; CTG: cytogenetics; FISH: Fluorescent in-situ hybridization; LEN: lenalidomide; BTZ: bortezomib; CFZ: carfilzomib; POM: pomalidomide; ref: refractory; unk: unknown.

### RT-PCR and qPCR

cDNA was prepared from RNA using the ABI high capacity cDNA kit (Thermo Fisher Scientific, Waltham, MA). qPCR was performed using TaqMan gene expression master mix (ABI 4368814) with an ABI 9600 Fast thermocycler as previously described [62]. The following ABI probes were used (Thermo Fisher Scientific): BAG3 (Hs00188713_m1), CRYAB (Hs00157107_m1), DNAJB1 (Hs00428680_m1), DNAJC17 (Hs01118821_g1), HSF1 (Hs00232134_m1), HSP90AA1 (Hs00743767_sH), HSP90AB1 (Hs01546471_g1), HSPA1A (Hs00359163_s1), HSPA1B (Hs01040501_sH), HSPB1 (Hs03044127_g1), HSPH1 (Hs00971475_m1) and GAPDH (Hs02758991_g1). For the 84-gene HSP expression array, the QIAGEN© Human Heat Shock Array qPCR Panel (PAHS-076C) was used according to manufacturer's instructions.

### Phos-Tag™

Protein lysates in 1X Protein MetalloPhosphatases (PMP) and 1X MnCl_2_ were treated with 64 units lambda (λ) phosphatase (New England Biolabs, Ipswich, MA) as per manufacturer's instructions. Protein was resolved on 50μM Phos-Tag™ (Wako Pure Chemical Industries, Osaka, Japan), 8% SDS-polyacrylamide gels as per manufacturer's instructions. Subsequent protein transfer and expression analysis was performed as described above.

### Immunoprecipitation and phosphoproteomics

Protein lysates were collected as described above. Lysates were precleared using Protein G Agarose, FastFlow (Millipore, Temecula, CA) as per manufacturer's instructions and antibody complex was formed using Preclearing Matrix B-rabbit: sc-45059 (Santa Cruz) and rabbit anti-HSF1 (Enzo) as per manufacturer's instructions. Precleared lysate was incubated with the antibody complex, and bound eluate was either resolved on a Mini-PROTEAN^®^ precast gel (Bio-Rad) and subsequently Coomassie stained (Bio-Rad) as per manufacturer's instructions, or the antibody complex was collected. Excised gel bands of interest or the antibody complex were sent to the Emory University School of Medicine Integrated Proteomics Core for liquid chromatography tandem mass spectrometry (LC-MS/MS) analysis (Supplementary Materials and Methods) [64].

### Immunocytochemistry

MM.1S cell pellets underwent formalin fixation and paraffin embedding. Immunostaining of cell block sections was performed essentially as described on a Dako autostainer [65]. Antigen unmasking employed Target Retrieval Solution citrate buffer (Dako). Anti-pS326-HSF1 was used at a 1:2000 dilution and bound antibody was detected with Envision dual link kit with standard DAB reactions (Dako). Hematoxylin counterstained sections were mounted for light microscopy.

## SUPPLEMENTARY METHODS - MASS SPECTROMETRY, FIGURE AND TABLE




