# Supplementary material for: Bortezomib-induced heat shock response protects multiple myeloma cells and is activated by heat shock factor 1 serine 326 phosphorylation
Source: Oncotarget. 2016 Jul 26;7(37):59727–41. doi: 10.18632/oncotarget.10847 (PMC5312344; doi:10.18632/oncotarget.10847)
Supplement: Supplementary file 1 [file oncotarget-07-59727-s001.pdf]

Bortezomib-induced heat shock response protects multiple myeloma cells and is activated by heat shock factor 1 serine 326 phosphorylation

SUPPLEMENTARY METHODS - MASS SPECTROMETRY

In-gel sample digestion

Gel bands were diced into ~1 mm cubes, destained with 50% acetonitrile (ACN) in 50 mM ammonium bicarbonate (ABC), dehydrated with ACN, and dried down using a SpeedVac (Thermo). Trypsin was added at a concentration of 10 ng/µL and samples were placed on ice for 30 minutes. The gel cubes were then covered with ABC buffer and digestion was allowed to proceed overnight. Peptides were extracted twice with 5% formic acid in 50/50 ABC/ACN solution. Each extraction consisted of 10 minutes of low vortexing in extraction buffer and 3 cycles of centrifugation with 1 min on and 1 min off per cycle. A final step of 100% ACN was used to extract all solution from the gel cubes and the entire peptide solution was dried completely by SpeedVac.

In-solution sample digestion

IP beads were resuspended in 50mM ammonium bicarbonate and treated with 1 mM dithiothreitol (DTT) at 25°C for 30 minutes, followed by 5 mM iodoacetimide (IAA) at 25°C for 30 minutes in the dark. Proteins were digested with 1 µg of lysyl endopeptidase (Wako) at room temperature for 2 hours and further digested overnight with 1:50 (w/w) trypsin (Promega) at room temperature. Resulting peptides were desalted with a Sep-Pak C18 column (Waters) and dried under vacuum.

LC-MS/MS orbitrap XL analysis

The dried peptides were resuspended in 10 µL of loading buffer (0.1% formic acid, 0.03% trifluoroacetic acid, 1% acetonitrile). Peptide mixtures (2 µL) were separated on a self-packed C18 (1.9 µm Dr. Maisch, Germany) fused silica column (15 cm x 75 µm internal diameter (ID); New Objective, Woburn, MA) by a double split liquid chromatography (LC) system consisting of an Agilent 1100 binary pump and a Famos autosampler. The LC system was interfaced to an Orbitrap XL mass spectrometer (ThermoFisher Scientific, San Jose, CA). Elution was performed over a 90 or 120 minute gradient at a rate of 300 nL/min (measured at the tip using a micropipette) with buffer B ranging from 3% to 80% (buffer A: 0.1% formic acid in water, buffer B: 0.1 % formic in acetonitrile). The mass spectrometer cycle was programmed to collect 1 precursor scan in the Orbitrap followed by 10 ion trap CID tandem (MS/MS) scans per cycle. The MS scans (300-1800 m/z range, 1,000,000 AGC, 150 ms maximum ion time) were collected at a resolution of 30,000 at m/z 200. Both the MS and CID MS/MS (2 m/z isolation width, 35% collision energy) scans were detected in centroid mode. Dynamic exclusion was set to exclude previous sequenced precursor ions for 20 seconds within a 10 ppm window.

LC-MS/MS Q-exactive analysis

The dried peptides were resuspended in 10 µL of loading buffer (0.1% formic acid, 0.03% trifluoroacetic acid, 1% acetonitrile). Peptide mixtures (2 µL) were separated on a self-packed C18 (1.9 µm Dr. Maisch, Germany) fused silica column (15 cm x 75 µm internal diameter (ID); New Objective, Woburn, MA) by a NanoAcquity UPLC (Waters) and monitored on a Q-Exactive Plus mass spectrometer (ThermoFisher Scientific, San Jose, CA). Elution was performed over a 90 minute gradient at a rate of 300nl/min with buffer B ranging from 3% to 80% (buffer A: 0.1% formic acid in water, buffer B: 0.1% formic acid in acetonitrile). The mass spectrometer cycle was programmed to collect 1 precursor scan followed by 10 HCD tandem (MS/MS) scan per cycle. The MS scans (300-1800 m/z range, 1,000,000 AGC, 150 ms maximum ion time) were collected at a resolution of 70,000 at m/z 200 in profile mode and the HCD MS/MS spectra (2 m/z isolation width, 30% collision energy, 50,000 AGC target, 50 ms maximum ion time) were detected at a resolution of 17,500 at m/z 200 in centroid mode. Dynamic exclusion was set to exclude previous sequenced precursor ions for 30 seconds within a 10 ppm window. Precursor ions with +1, and +6 or higher charge states were excluded from sequencing.

Database search parameters

Spectra were searched using the same parameters on one of two software programs (A) Spectra were searched using Sequest Sorcerer version 4.3 (Sage-N Research) against a decoy supplement human REFSEQ database (version 62 with 68,742 target sequences). Searching parameters included fully tryptic restriction and a parent ion mass tolerance (± 50 ppm). Methionine oxidation (+15.99492Da) and serine, threonine, and tyrosine phosphorylation (+79.966331Da) were variable modifications (up to 3 allowed per peptide); cysteine

was assigned a fixed carbamidomethyl modification (+57.021465 Da). The peptides were classified by charge state and filtered dynamically by increasing XCorr and ΔCn values to reduce protein false discovery rate to less than 1%, according to the target-decoy strategy. (B) Spectra were searched using Proteome Discoverer 1.4 against a decoy supplement human REFSEQ database (version 62 with 68,742 target sequences). Searching parameters included fully tryptic restriction and a parent ion mass tolerance (± 50 ppm). Methionine oxidation (+15.99492Da) and serine, threonine, and tyrosine phosphorylation (+79.966331Da) were variable modifications (up to 3 allowed per peptide); cysteine was assigned a fixed carbamidomethyl modification (+57.021465 Da). The peptide matches were filtered in using percolator to a psm level fdr of 1%.

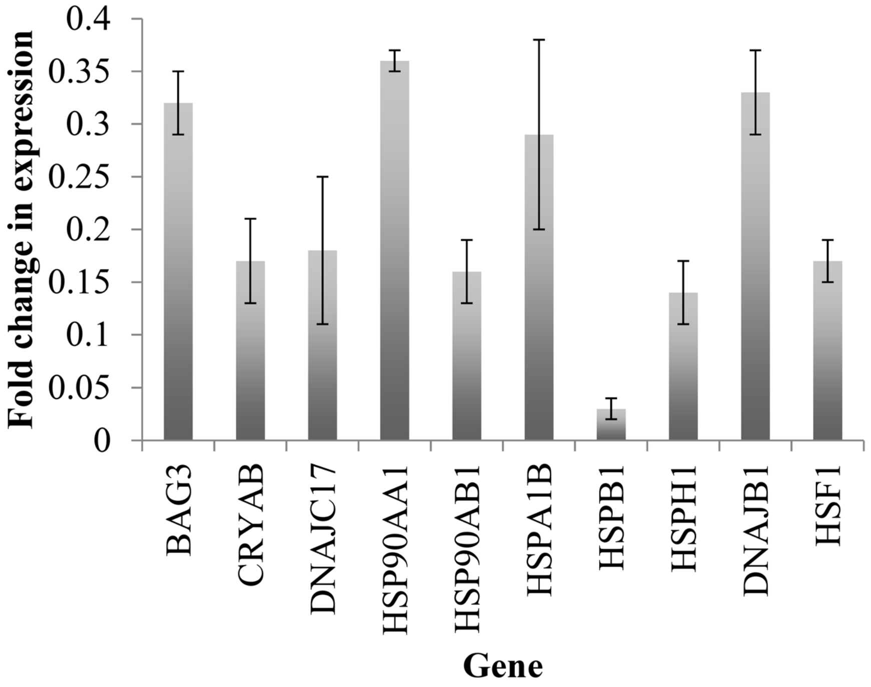

Supplementary Figure S1: HSP or HSF1 silencing leads to robust knockdown 48h after transfection. MM.1S cells were treated with a non-silencing control [si(-)] or HSP or HSF1 siRNA for 24h followed by 0 (untreated) or 4 nM bortezomib for an additional 24h. Gene expression is shown for untreated cells relative to si(-) and normalized to GAPDH endogenous control. Data are presented as the mean±s.e. of three independent experiments.

Supplementary Table S1: Numeric values for the fold change in gene expression represented in Figure 2A. Data are presented as the fold change compared to MM.1s cells transfected with the non-silencing control siRNA (-) and treated for zero hours with bortezomib. (+) indicates transfection with siHSF1 and 4 indicates hours of bortezomib treatment**.**

See Supplementary File 1
